# Preparation of phase change material filled hybrid 2D/3D graphene structure with ultra-high thermal effusivity for effective thermal management

**DOI:** 10.1016/j.mex.2021.101385

**Published:** 2021-05-15

**Authors:** Gengyuan Liang, Jianwei Zhang, Shaohang An, Jun Tang, Su Ju, Shuxin Bai, Dazhi Jiang

**Affiliations:** a Department of Materials Science and Engineering, National University of Defense Technology, No. 109, Deya Road, Changsha 410073, PR China; b Guangzhou Higher Education Mega Center, Sun Yat-Sen University, No. 132, Waihuan East Road, Guangzhou 510006, PR China

**Keywords:** Graphene, Thermal management, Energy storage

## Abstract

Graphene-based energy storage and renewable material has increasingly attracted research interest, due to its high thermal conductivity and light weight. Researchers fill phase change material (PCM) into three-dimensional graphene foam, to obtain a composite with high energy storage capability and moderate thermal conductivity. However, this kind of composite's heat transfer mode is single and cannot maximize the advantages of graphene. Herein, a stearic acid filled graphene-foam composite (GFSAC) connected with graphene paper (GP) through gravity-assisted wetting attaching process is demonstrated in this paper.

• GP is obtained by thermal reduction of graphene oxide (GO) paper. Its in-plane thermal conductivity can reach up to 938 Wm^−1^ K^−1^. By controlling the preparation process of GO paper, the in-plane thermal conductivity of GP can be adjusted.
• GFSAC is consisted of GF and SA, GFSAC with different heat transfer properties can be prepared by adjusting the degree of reduction of GF.
• A novel gravity-assisted wetting attaching process has been developed to prepare GP/GFSAC/GP composite, which can effectively reduce the thermal resistance between GP and GFSAC.

The effective thermal effusivity of the final GP/GFSAC/GP composite reaches 18.45 J cm^−3/2^ m^−1/2^ s^−1/2^ K^−1/2^, showing an excellent thermal management capability.

## Graphical abstract

**Figure:**
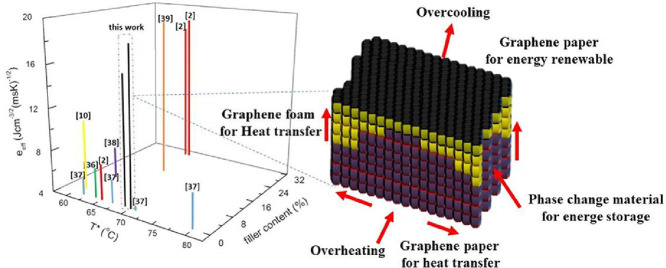

Specifications table

**Table:** 

Subject Area:	Energy
More specific subject area:	Thermal management for electronics devices to prevent overheating and overcooling
Method name:	gravity-assisted wetting attaching process
Name and reference of original method:	[1] G. Liang, J. Zhang, S. An, J. Tang, S. Ju, S. Bai, D. Jiang, Phase change material filled hybrid 2D/3D graphene structure with ultra-high thermal effusivity for effective thermal management, [CARBON_15957]. [2] S. Lin, S. Ju, G. Shi, J. Zhang, Y. He, D. Jiang, Ultrathin nitrogen-doping graphene films for flexible and stretchable EMI shielding materials, J. Mater. Sci. 54 (2019) 7165–7179. https://doi.org/10.1007/s10853-019-03372-4. [3] J. Zhang, G. Shi, C. Jiang, S. Ju, D. Jiang, 3D Bridged Carbon Nanoring/Graphene Hybrid Paper as a High-Performance Lateral Heat Spreader, Small. 11 (2015) 6197–6204. https://doi.org/10.1002/smll.201501878. [4] X. Xu, C. Guan, L. Xu, Y.H. Tan, D. Zhang, Y. Wang, H. Zhang, D.J. Blackwood, J. Wang, M. Li, J. Ding, Three Dimensionally Free-Formable Graphene Foam with Designed Structures for Energy and Environmental Applications, ACS Nano. 14 (2020) 937–947. https://doi.org/10.1021/acsnano.9b08191.
Resource availability:	*The GO aqueous solution is purchased from GaoxiTech company, Hangzhou, China. The SA is purchased from Xinkang company, Tianmen, China. The heat-conducting glue is KP98, produced by Kerafol, Germany.*

## Method details

In the final GP/GFSAC/GP composite, high thermal conductivity of 1.72 Wm^−1^ K^−1^ (with 0.53 wt% graphene loading), and excellent effective thermal effusivity of 18.45 J cm^−3/2^ m^−1/2^ s^−1/2^ K^−1/2^ has been achieved.

### Preparation of graphene paper (GP)

**Fig. 1:**
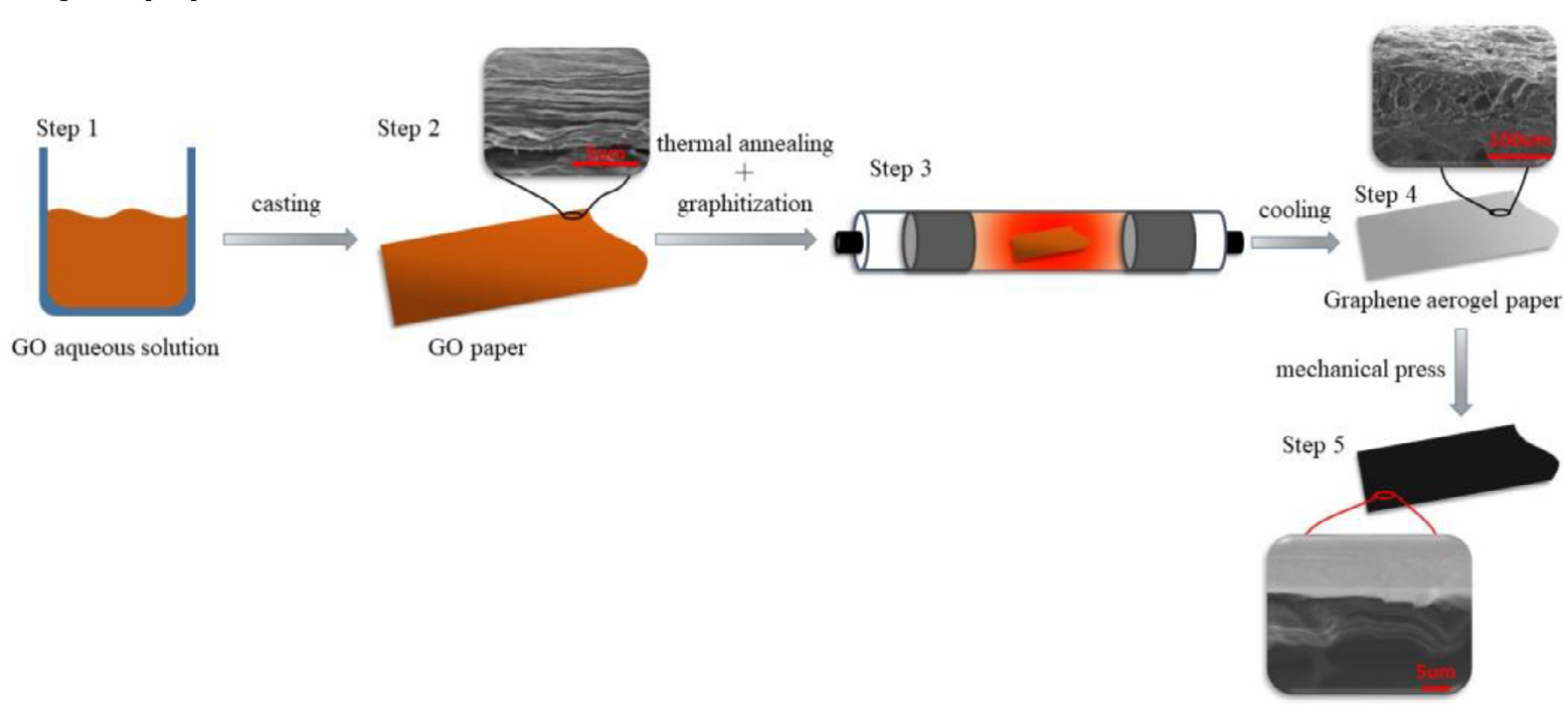
Preparation process of GP.

The GP is prepared by annealing graphene oxide (GO) paper at 2600 °C. Firstly, we used 8 mg/ml GO aqueous solution to prepare GO paper on copper foil through casting method. And the thickness of the paper can be effectively controlled by adjusting the moving speed of the blade and the distance between the blade and the copper foil. Then, the GO paper was reduced to GF through a two-step thermal annealing method, first annealed at 1100 °C to get reduced graphene oxide (rGO) paper, and then graphitized at 2600 °C to obtain GP. In order to improve the density and thermal conductivity of GP, the GP obtained by annealing was pressed at 20 MPa for 5 min, the preparation process is shown in Fig. 1 [1], [2], [3].

### Preparation of stearic acid filled graphene-foam composite (GFSAC)

**Fig. 2:**
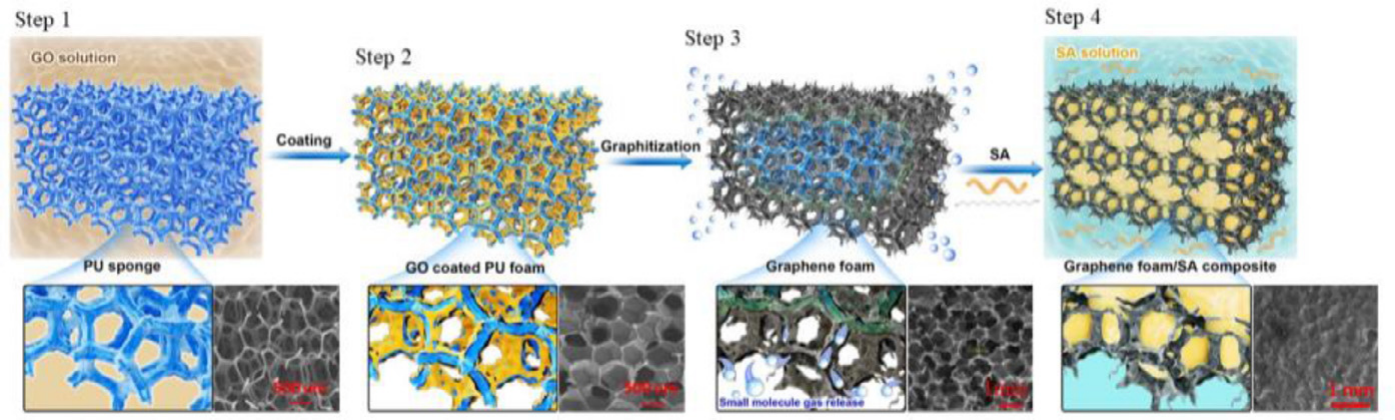
Preparation process of GFSAC.

Firstly, polyurethane (PU) foam was chosen as the template and repeatedly impregnated in the GO aqueous solution under vacuum [4]. Next, the two-step thermal annealing method was also used to remove the PU and reduce the GO foam to GF at the same time. Finally, the GF was fully impregnated into SA under vacuum to prepare GFSAC, the preparation process is shown in Fig. 2.

### Preparation of GP/GFSAC/GP composite

**Fig. 3:**
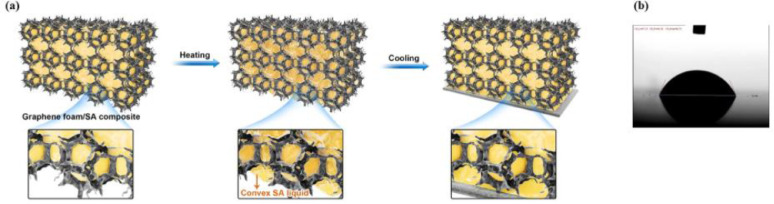
Preparation process of GP/GFSAC/GP composite. a) Combine of GP and GFSAC to fabricate GP/GFSAC/GP composite, and the insets illustrate the mechanism of this process. b) The contact angle between GP and SA liquid.

In detail, as shown in Fig. 3a, a piece of GP is placed at the bottom of the GFSAC and the structure is heated to the melting point of SA under vacuum condition. When SA is melted, the SA liquid in GFSAC will have a downward penetration tendency under the influence of gravity. The liquid SA will enrich at the bottom of the GFSAC, forming a convex liquid level, as shown in the insets of Fig. 3a. Once the liquid SA contacts GP at the bottom of GFSAC, the capillary force will drive SA liquid to fill the gap between GP and GFSAC. The good wetting properties between SA and GP (the average wetting angle is 70.06°, as shown in Fig. 3b), will allow SA liquid to fully wet GP.

### Method validation

**Fig. 4:**
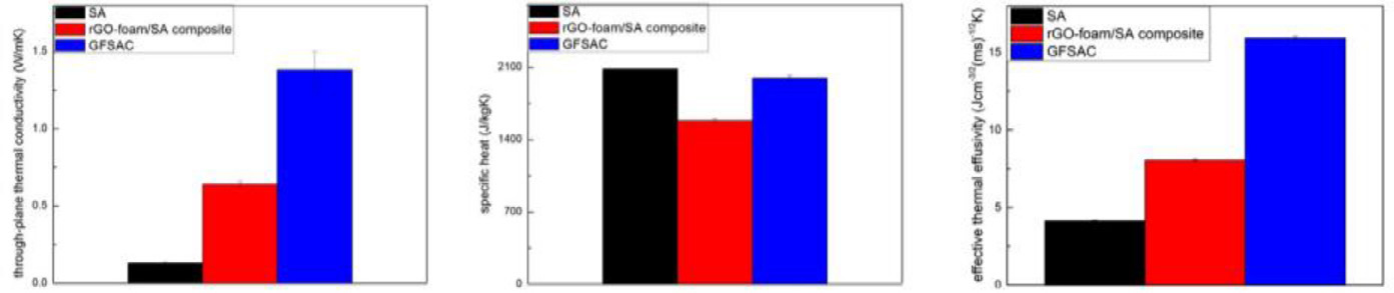
Through-thickness thermal conductivity, specific heat and effective thermal effusivity of SA, rGO-foam/SA composite and GFSAC.

1100 °C annealed rGO-foam and 2600 °C annealed GF were used to prepare the rGO-foam/SA composite and GFSAC. For GFSAC, the mass fraction of graphene is only 0.48 %, while its thermal conductivity increases to 1.38 Wm^−1^ K^−1^ for GFSAC at RT, as shown in Fig. 4. Although with the addition of GF, the specific heat and phase change enthalpy of the GFSAC have a little decrease, the composite's *e_eff_* is greatly improved from 4.15 Jcm^−3/2^ m^−1/2^ s^−1/2^ K^−1/2^ for SA to 15.93 Jcm^−3/2^ m^−1/2^ s^−1/2^ K^−1/2^, illustrating the GFSAC possesses outstanding thermal energy storage capacity. This result proves that the GF skeleton plays an irreplaceable role in heat conduction and storage for this composite, although the mass fraction of graphene is very low.

**Fig. 5:**
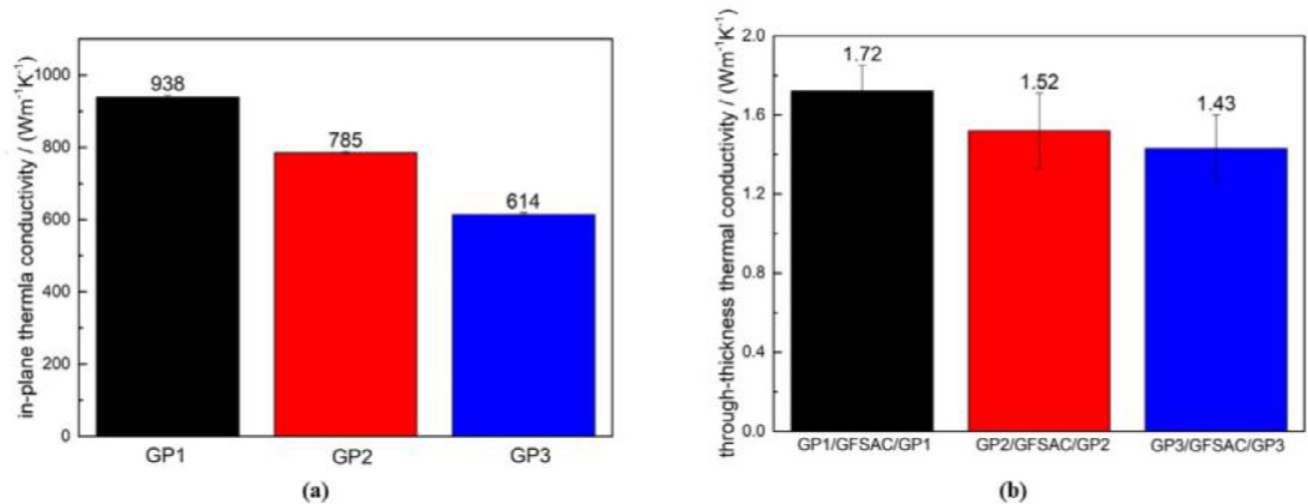
Thermal conductivity test. a) In-plane thermal conductivity of GP1, GP2 and GP3. b) Through-thickness thermal conductivity of GP1/GFSAC/GP1, GP2/GFSAC/GP2 and GP3/GFSAC/GP3.

In order to explore the synergy between 2D GP and 3D GFSAC on thermal conductivity mechanism, GPs with different thicknesses of 3 μm, 8 μm and 13 μm (measured by micrometer screw gauge) have been fabricated, numbered as GP1, GP2 and GP3. As the thickness increases, the thermal conductivity of GP decreases gradually, as shown in Fig. 5a. Next, GP1/GFSAC/GP1, GP2/GFSAC/GP2 and GP3/GFSAC/GP3 have been prepared, and their through-thickness thermal conductivity has been tested, as shown in Fig. 5b. With the increasing of the in-plane thermal conductivity of the 2D GP, the through-thickness thermal conductivity of GP/GFSAC/GP composite also increases, indicating that rapid in-plane heat conduction will indeed shorten the time for the structure temperature to reach equilibrium and improve the thermal management capacity.

**Fig. 6:**
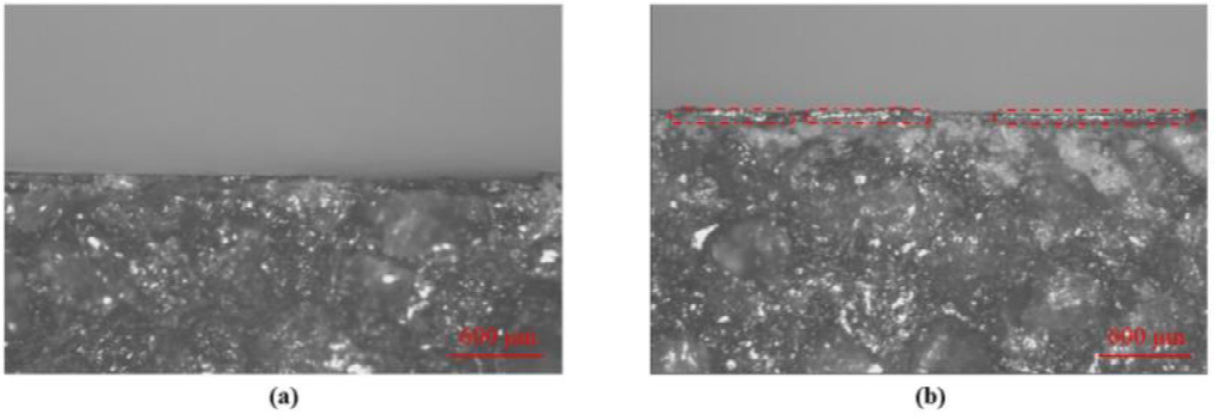
Optical microscope image of the banding phase of G-GP/GFSAC/GP composite and GP/GFSAC/GP composite. a) GP/GFSAC/GP composite. b) G-GP/GFSAC/GP composite.

In order to verify the role of gravity in the molding process, heat-conducting glue is also used to connect GP and GFSAC, and the glue connected GP/GFSAC/GP (G-GP/GFSAC/GP) composite is prepared as a comparison. Interestingly, though the heat-conducting glue has higher thermal conductivity (6 Wm^−1^ K^−1^) than that of SA (0.13 Wm^−1^ K^−1^), the through-thickness thermal conductivity of G-GP/GFSAC/GP composite is only 0.53 Wm^−1^ K^−1^, which is much lower than that of GP/GFSAC/GP composite. The interface between GP and GFSAC of GP/GFSAC/GP and G-GP/GFSAC/GP is further characterized, and shown in Fig. 6. It can be found that the banding phase of G-GP/GFSAC/GP composite is thicker than that of GP/GFSAC/GP composite. In GP/GFSAC/GP, GP is almost directly connected to GFSAC, while in G-GP/GFSAC/GP, the banding phase can be clearly observed between GP and GFSAC. In a conclusion, through gravity-assisted wetting attaching process, not only the GP and GFSAC can be connected tightly, but the composite can possess higher through-thickness thermal conductivity than that of the composite prepared through other process as well.

## Declaration of Competing Interest

The authors confirm that there are no conflicts of interest.
